# Optimal features assisted multi-attention fusion for robust fire recognition in adverse conditions

**DOI:** 10.1038/s41598-025-09713-5

**Published:** 2025-07-04

**Authors:** Inam Ullah, Nada Alzaben, Yousef Ibrahim Daradkeh, Mi Young Lee

**Affiliations:** 1 Department of Computer Engineering, Gachon University, Seongnam, 13120 South Korea; 2 Department of Computer Sciences, College of Computer and Information Sciences, Princess Nourah Bint Abdulrahman University, 11671 Riyadh, Saudi Arabia; 3 Department of Computer Engineering and Information, College of Engineering in Wadi Alddawasir, Prince Sattam bin Abdulaziz University, 16273 Al-Kharj, Saudi Arabia; 4 Office of the Research, Chung-Ang University, Seoul, South Korea

**Keywords:** Fire detection, Explainable AI, Visual sensors, Attention mechanisms, Deep learning, Edge computing, Engineering, Mathematics and computing

## Abstract

Deep neural networks have significantly enhanced visual data-based fire detection systems. However, high false alarm rates, shallow-layered networks, and poor recognition in challenging environments continue to hinder their practical deployment. To address these limitations, we introduce the Attention-Enhanced Fire Recognition Network (AEFRN). This novel progressive attention-over-attention framework achieves state-of-the-art (SOTA) performance while maintaining computational efficiency. Our approach introduces three key innovations: Firstly, Convolutional Self-Attention (CSA), integrating global self-attention with convolution through dynamic kernels and trainable filters for enhanced low-level fire feature processing. Secondly, Recursive Atrous Self-Attention (RASA) with optimized dilation rates, capturing comprehensive multi-scale contextual information through a recursive formulation with minimal parameter overhead. Thirdly, an enhanced Convolutional Block Attention Module (CBAM) with modified channel and spatial attention mechanisms for robust feature discrimination. We validate AEFRN’s interpretability using Grad-CAM visualization, demonstrating effective attention focus on fire-relevant regions. Comprehensive experimental evaluation on FD and BoWFire benchmark datasets shows AEFRN’s superiority over SOTA methods, achieving 99.11% accuracy on the FD dataset, and 97.98% accuracy on the BoWFire dataset. Extensive comparisons against twelve SOTA approaches confirm AEFRN’s effectiveness for fire detection in challenging scenarios while maintaining computational efficiency suitable for practical deployment.

## Introduction

Fire is the most dangerous and destructive catastrophe due to its rapid proliferation and disastrous environmental consequences. The efficient management of fire is a challenging task, especially in areas with a high intensity of combustible materials, such as forests^1^, residential zones^2^, and many other sensitive environments^3^. Fire can arise from several causes involving human activities^4^, machinery failures^5^, rising temperatures, and climate change^6^. Forest and bushfires, considered the most hazardous kind of fire, may cause significant harm to the environment due to their rapid expansion. These flames pose a substantial danger since their intensity rapidly grows, raising the possibility of widespread ecological damage. Recently, around 19 million hectares of land were burned down by destructive bushfires in Australia in the first quarter of 2020^7^. Unfortunately, it decimated over 3,000 residences and caused the loss of over 1.5 billion animal lives, resulting in a path of grief and devastation^8^. In 2021, the US Fire Administration documented 353,500 residential fires, resulting in 2,840 fatalities, 1,400 injuries, and $8.86 billion in financial damages^3^. In light of these challenges, researchers have proposed various fire detection techniques that depend on visual or environmental sensors. Early fire recognition is crucial in reducing the loss of human life and limiting other types of damage. Soft computing approaches, which use multiple sensory input sources, are becoming an effective option for enabling prompt detection and response.

Vision-based fire recognition systems can be generally classified into traditional machine learning (ML) and deep learning (DL) techniques. Conventional ML-based approaches focus on analyzing fire shape, texture, color, and motion features^9–12^. The effectiveness of these techniques heavily depends on traditionally obtained features. However, selecting the most suitable features poses a considerable challenge. Factors such as the changing shape of fire due to airflow, the influence of lighting conditions, and variations in fire colors across different materials make it challenging to balance false positive rate, loss, and overall accuracy. Achieving an optimal balance among fire detection metrics presents a persistent challenge in conventional detection methods. DL has revolutionized fire detection, garnering extensive recognition and integration in numerous applications. However, many existing datasets are limited in diversity, generally consisting of only two classes: fire and non-fire. Even with its advantages, DL models encounter specific challenges when handling complex fire scenes.

Identifying fires in sunlight that resemble flames, variations in lighting that reflect fire-like colors, and objects that visually imitate fire present substantial challenges^13,14^. These complex scenarios expose critical limitations in existing approaches, which often lack the discriminative capability to differentiate between actual fire incidents and visually similar elements. A fundamental challenge to advancing fire detection research is the inadequate representation of real-world complexities in existing datasets, as highlighted in^15^. Moreover, while advanced DL models have shown promise, their deployment in practical surveillance systems faces significant constraints due to computational overhead and resource limitations^16,17^. Additionally, existing attention modules typically operate independently without considering the hierarchical relationships between different attention mechanisms. This often leads to suboptimal feature representation and reduced detection accuracy in challenging scenarios. The lack of effective attention modules that can simultaneously address spatial localization, channel-wise feature enhancement, and multi-scale contextual understanding represents a critical gap in current literature. These limitations necessitate the development of attention-based networks that can effectively integrate multiple attention mechanisms for effective detection in challenging visuals.

To address these limitations, we introduce the Attention-Enhanced Fire Recognition Network called AEFRN. The proposed network combines the potential of Convolutional Self-Attention (CSA), Recursive Atrous Self-Attention (RASA), and an enhanced Convolutional Block Attention Module (CBAM) through a hierarchical attention-over-attention paradigm. Our network shows the capability to simultaneously extract detailed local fire features and scene-level information via its adaptive multi-resolution attention mechanism. The CSA module integrates traditional convolution operations with global self-attention mechanisms to focus on fundamental fire-specific visual patterns. RASA uses strategically configured atrous convolutions with optimized dilation parameters to capture contextual information across different spatial scales. Additionally, we present an optimized CBAM architecture featuring strengthened channel-wise and spatial attention components that enhance the network’s capacity for fire-relevant features. This unified attention framework facilitates robust fire detection performance under challenging operational conditions, such as partial occlusions, dynamic lighting variations, and environments with visually similar non-fire objects. It showcases state-of-the-art (SOTA) performance across established benchmark datasets.

### Contributions

The main contributions of this research are outlined as follows:

- We introduce AEFRN, a hierarchical attention framework that effectively combines CSA, RASA, and an improved CBAM using a layered attention-over-attention approach. Our CSA component seamlessly integrates global self-attention mechanisms with spatially-focused convolution operations by utilizing adaptive kernel configurations and trainable filter parameters, which allow for the robust identification of essential fire-specific visual patterns. The RASA architecture implements thoughtfully designed multi-scale dilated convolutions with optimized dilation coefficients to gather comprehensive contextual representations across different spatial ranges.
- We propose a novel MCBAM that incorporates sophisticated channel-wise and spatial attention components. This enhanced architecture systematically improves feature representation quality by amplifying fire-associated spatial regions and feature channels, while simultaneously suppressing noise from background distractors and visually similar non-fire elements. The combined attention modules operate cohesively, delivering improved feature selectivity without imposing substantial computational cost.
- Extensive ablation experiments are carried out to validate each component of the proposed framework. These include an analysis of backbone networks, where ResNet50 demonstrates the best performance; evaluations of various attention module combinations, showing the complementary strength of CSA, RASA, and MCBAM; and optimization of data augmentation strategies to boost model generalization across diverse fire detection scenarios. The studies also validate the optimal dilation rate configuration and demonstrate that AEFRN’s computational efficiency is suitable for edge device deployment.
- We achieve SOTA performance on benchmark datasets, with AEFRN attaining 99.11% accuracy and 98.88% F1-score on the FD dataset, and 97.98% accuracy and 97.94% F1-score on the BoWFire dataset. These results represent significant improvements over existing methods, including MAFire-Net and ConvNeXt-TSA, demonstrating the effectiveness of our progressive attention framework for practical fire detection applications.

The forthcoming sections of this paper are organized as follows: Section 2 presents a comprehensive literature review, providing an overview of traditional ML and DL-based methods along with hybrid approaches. In Section 3, we elaborate on the details of our proposed methodology. Section 4 presents and discusses the datasets used for performance evaluation, parameter settings, comparative analysis, ablation study, model complexity, and detailed results. Finally, in Section 5, we conclude the paper by providing insights into potential directions for future research.

## Related work

Extensive research is underway in fire detection, with a focus on computer vision (CV)-based networks. This automation reduces the need for human intervention, enabling swift response times. These advanced techniques leverage CV algorithms to achieve early and accurate fire detection. Fire detection approaches can be broadly classified into two main categories: 1) Traditional ML-based approaches and 2) DL-based methods.

### Machine learning based approaches

ML-based fire detection techniques leverage various image attributes, including color, texture, motion, and shape. Baseline techniques^12,18–22^ utilize the YCbCr and RGB color spaces to extract color features for fire detection. The literature extensively incorporates fuzzy logic, superpixel texture discrimination, and statistical color features in an integrated fashion^9,23,24^. Furthermore, fire recognition research frequently examines moving objects using optical flow features. These ML techniques, however, are often susceptible to environmental influences, such as dynamic entities resembling fire and objects with orange or red hues, which contribute to increased false positive rates. The conventional approach of maintaining brightness consistency fails to accurately capture the characteristics of fire. Additionally, optical flow-based methods require substantial computational resources. To mitigate the subjective impact on the recognition process, researchers have focused on trainable classifiers. Works in^25,26^ propose utilizing unimodal Gaussian covariance features extracted from spatial-temporal regions and employing support vector machines (SVM) for classifying areas exhibiting fire-like colors in motion. Optimizing feature selection for immediate recognition of fire scenes and generating accurate alarms presents considerable challenges in ML-based methods. These challenges stem from factors such as variations in shiny environments and similar moving elements like fire and shadows, which can degrade model performance. Consequently, numerous researchers have investigated end-to-end DL-based methods to address these challenges and improve fire scene classification effectiveness.

### Deep learning based approaches

DL has emerged as a prominent data-driven approach, finding extensive applications across diverse fields^27,28^. Its efficiency extends to various tasks, including classification, detection, and segmentation. With its ability to learn intricate patterns and representations from data, DL has demonstrated formidable prowess in tackling complex challenges across multiple domains. Several researchers have investigated Convolutional Neural Networks (CNNs) for fire detection to address the limitations inherent in ML techniques^29–31^. CNN models provide a significant advantage through their ability to extract and recognize features efficiently, enhancing convenience and reliability. For instance, work in^32^ assessed fire recognition efficiency using AlexNet, VGG, and GoogLeNet networks, with GoogLeNet yielding the most favorable outcomes. Similarly, another study^33^ introduced ResNet50 and VGG16 networks, where ResNet50 demonstrated superior performance compared to VGG16. However, their experimental dataset was limited in size, potentially impacting the generalizability of their findings. Previous studies^19–28,32,33^ using LeNet-5 and AlexNet models (plain CNN architectures) demonstrated superior performance in fire scene classification compared to ML-based approaches. Nonetheless, these models are unsuitable for resource-constrained settings due to their computational complexity and large model sizes. Recognizing this limitation, the work in^34^ investigated a lightweight CNN network known as InceptionV1. Moreover, Khan et al. achieved improved results by reducing model size and time complexity while examining various CNN-based models.

Many researchers explored impactful support measures, including detection of combustible materials, evacuation observation, counting, and target type detection. Current research on implementing DL based methods on resource-constrained devices (RCDs) primarily focuses on two key concerns: reducing model complexity and size. Various model compression techniques have been investigated, including weight pruning and weight quantization^35^. Pruning involves removing unstructured neurons, filters, and weights from the network. This can be achieved through salience ranking (either in a single step or through multiple refinement iterations) and by utilizing sacrificial norms^36,37^. Conversely, quantization encompasses a broader research scope involving low-precision weights and activation functions during network training^38,39^. These techniques also leverage specialized hardware to enhance real-time inferencing capabilities^40^. Through model compression methods and hardware optimizations, researchers aim to overcome the challenges associated with implementing DL-based approaches in real-time on edge devices while simultaneously reducing model complexity and size. Furthermore, researchers have attempted to combine ML with CNNs to achieve effective fire scene recognition. The work in^41^ combined SVM with CNN for fire detection, utilizing an AdaBoost cascade with Haar features to extract regions of interest. They implemented a shallow CNN containing four straightforward network layers for feature extraction and an SVM for fire recognition. Authors in^42^ combined CNN with motion detection to analyze irregularities for smoke and fire recognition. Another study^43^ proposed a real-time method merging local binary patterns and AdaBoost for extracting regions of interest, employing CNN for feature extraction from the extracted regions, followed by classification. The work in^44^ proposed spatial and temporal feature-assisted networks for fire recognition in complex environments. Additionally, attention-based mechanisms have been integrated with CNN architectures across various domains to enhance model performance^45,46^. These mechanisms offer promising results by selecting optimal features before classification, encouraging their utilization for accurate fire view recognition^15,47–49^. However, the literature demonstrates that there remains room for exploration in developing robust and accurate fire scene classification and localization models. Moreover, the robustness of proposed models is typically analyzed on limited datasets. Therefore, this study explores a computationally efficient network that effectively addresses fire scene recognition challenges when trained on diverse datasets.

## Proposed methodology

**Fig. 1:**
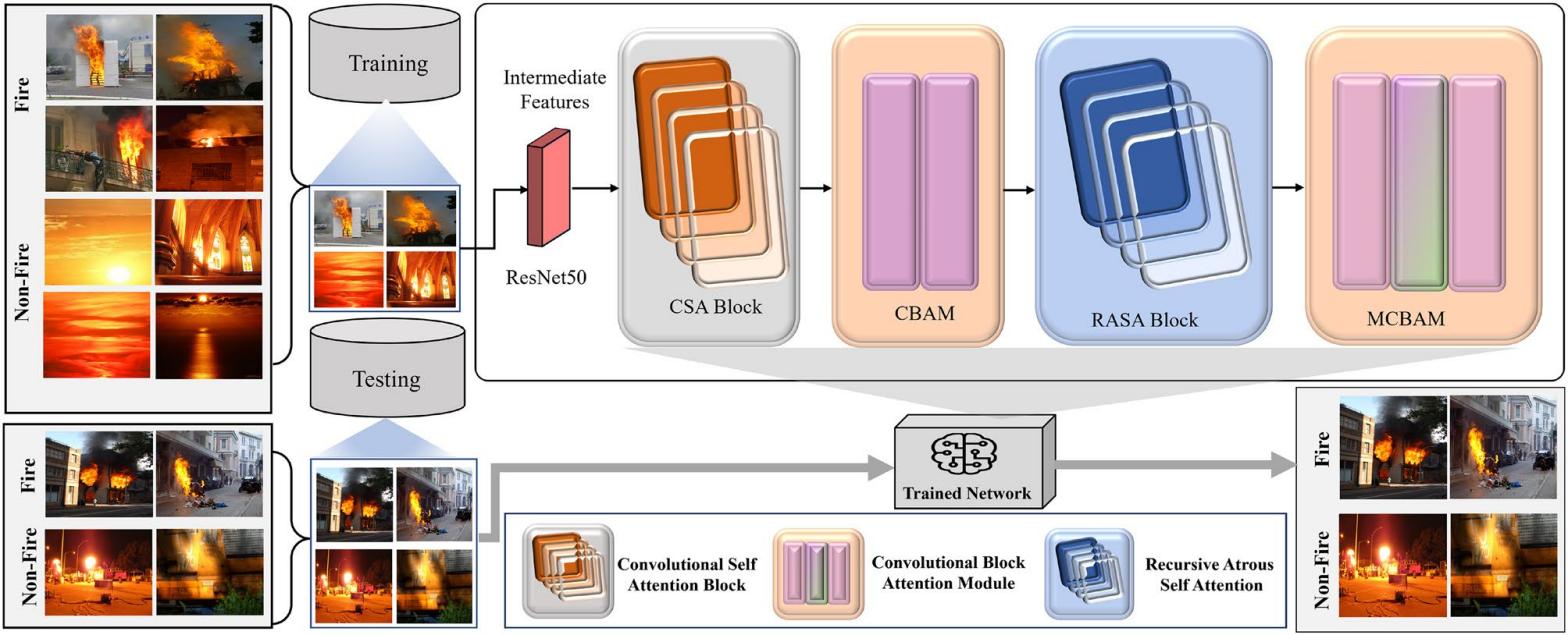
Proposed fire detection network architecture showing the complete pipeline: ResNet50 backbone extracts semantic features from input images, followed by sequential processing through CSA for local-global context modeling, CBAM for spatial and channel-wise attention refinement, RASA for multi-scale feature enhancement, and MCBAM for final feature optimization, demonstrating the progressive attention-over-attention framework for effective fire detection.

The proposed network comprises a systematic five-stage architecture for effective fire detection as illustrated in Fig. 1, following the pipeline: ResNet50 $\rightarrow$ CSA $\rightarrow$ CBAM $\rightarrow$ RASA $\rightarrow$ MCBAM $\rightarrow$ Classification. The architecture begins with a ResNet50 backbone processing $256 \times 256 \times 3$ input images to extract $8 \times 8 \times 2048$ semantic features from conv5_x output, which serve as the foundation for subsequent attention-based processing. The CSA module then utilizes $3 \times 3$ depthwise convolutions followed by multi-head self-attention to integrate local feature enhancement with global context modeling across the 64 spatial locations. Following CSA, a modified CBAM applies channel and spatial attention mechanisms, with the key innovation of replacing traditional $7 \times 7$ convolutions with efficient sequential $1 \times 1$ and $3 \times 3$ convolutions in the spatial attention component, culminating with Global Average Pooling for comprehensive feature refinement. The RASA module then combines Atrous Self-Attention with recursive formulation, utilizing atrous convolutions with dilation rates $\{1, 3, 5\}$ to capture multiscale context within self-attention calculations, where the recursive depth is limited to two iterations for computational efficiency. Finally, Modified CBAM (MCBAM) operates on the multi-scale enhanced features from RASA, maintaining the same $1 \times 1$ + $3 \times 3$ architectural improvements to perform final feature optimization and prioritize the most significant regions for accurate fire detection. This progressive attention-over-attention framework creates a comprehensive feature enhancement pipeline where each stage builds upon the previous outputs, resulting in increasingly refined and contextually aware representations optimized for challenging fire detection scenarios.

### Deep features extraction

Recent studies have emphasized the significance of integrating effective intermediate features from earlier convolutional stages^50,51^. They maintain essential spatial and structural details that are critical for improving downstream tasks. To achieve strong feature representation, we utilize ResNet50 as our backbone network for extracting high-level semantic features from input images. With its deep residual architecture, ResNet50 has shown remarkable effectiveness in capturing hierarchical visual representations across a range of computer vision tasks. The ResNet50 backbone processes input images of size $256 \times 256 \times 3$ through a series of convolutional blocks with residual connections. The network architecture consists of an initial convolutional layer followed by four main residual blocks (conv2_x, conv3_x, conv4_x, and conv5_x), each containing multiple bottleneck residual units. The residual connections in ResNet50 enable effective training of deep networks by mitigating the vanishing gradient problem and facilitating information flow across layers. We utilize the output from the final convolutional block (conv5_x) of ResNet50, which produces feature maps of dimensions $8 \times 8 \times 2048$. This choice is motivated by several factors: (i) the $8 \times 8$ spatial resolution provides a manageable number of spatial locations (64 tokens) for efficient attention computation; (ii) the 2048-dimensional feature vectors contain rich semantic representations learned through the deep hierarchical structure; and (iii) the features at this level capture high-level semantic information while maintaining spatial locality, making them ideal for subsequent attention-based processing.

The mathematical formulation for ResNet50 feature extraction can be expressed as:

1 $$\begin{aligned} {\textbf{F}} = \text {ResNet50}({\textbf{I}}) \end{aligned}$$

where ${\textbf{I}} \in {\mathbb {R}}^{B \times 3 \times 256 \times 256}$ represents the input batch of images and ${\textbf{F}} \in {\mathbb {R}}^{B \times 2048 \times 8 \times 8}$ denotes the extracted deep features.

**Fig. 2:**
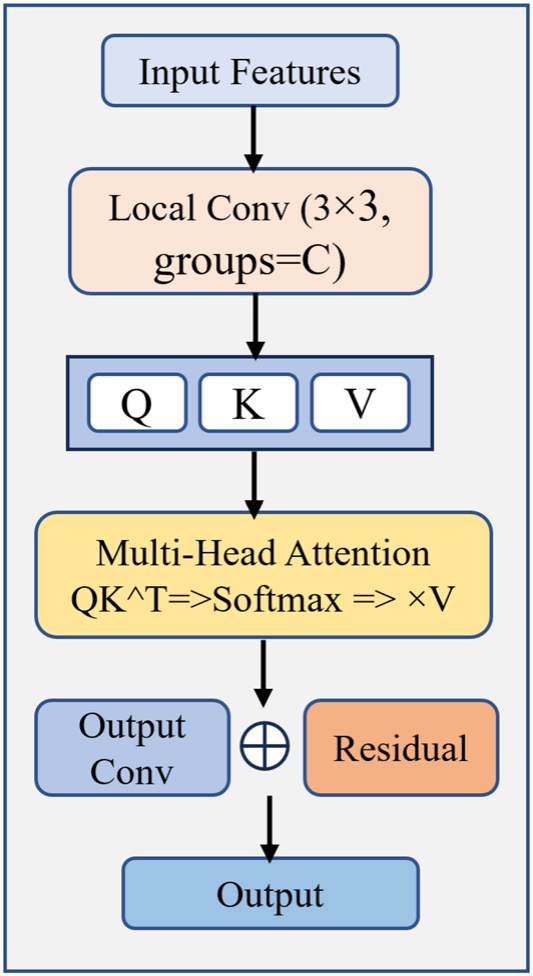
Architecture of the CSA module showing the sequential processing of ResNet50 features for enhanced feature representation.

### Convolutional self-attention (CSA) module

Building upon the deep features extracted by ResNet50, we proposed a novel CSA module that effectively combines local feature enhancement with global context modeling. The CSA module is utilize the high-dimensional semantic features (${\textbf{F}} \in {\mathbb {R}}^{B \times 2048 \times 8 \times 8}$) generated by ResNet50. It improves their representational power through attention mechanisms while ensuring computational efficiency. Unlike conventional self-attention that directly processes input features, our CSA module first conducts local convolutions to identify spatial patterns, subsequently calculating attention weights. This approach yields more robust and contextually informed feature representations. By leveraging the rich semantic information inherent in ResNet50 features, it also enhances their capacity for modeling long-range dependencies. As shown in Fig. 2, the CSA module comprises six key components that collaboratively enhance the feature representations of ResNet50.

**Local Context Extraction:** The ResNet50-extracted feature map ${\textbf{F}} \in {\mathbb {R}}^{B \times 2048 \times 8 \times 8}$ first passes through a depthwise separable convolution layer:

2 $$\begin{aligned} {\textbf{F}}_{\text {local}} = \text {DWConv}_{3 \times 3}({\textbf{F}}) \end{aligned}$$

This operation employs grouped convolutions, allowing each of the 2048 ResNet50 feature channels to be processed independently while preserving local spatial patterns in a $3 \times 3$ neighborhood. **Query, Key, Value Generation:** Following the local context extraction, three parallel $1 \times 1$ convolutions generate the query (Q), key (K), and value (V) matrices:

3 $$\begin{aligned} {\textbf{Q}}&= \text {Conv}_{1 \times 1}({\textbf{F}}_{\text {local}}) \end{aligned}$$

4 $$\begin{aligned} {\textbf{K}}&= \text {Conv}_{1 \times 1}({\textbf{F}}_{\text {local}}) \end{aligned}$$

5 $$\begin{aligned} {\textbf{V}}&= \text {Conv}_{1 \times 1}({\textbf{F}}_{\text {local}}) \end{aligned}$$

**Multi-Head Attention Computation:** The Q, K, V tensors are reshaped to enable multi-head attention processing with heads=4, resulting in head_dim = 512 and spatial tokens $H \times W = 64$:

6 $$\begin{aligned} \text {Attention}({\textbf{Q}},{\textbf{K}},{\textbf{V}}) = \text {Softmax}\left( \frac{{\textbf{Q}}{\textbf{K}}^T}{\sqrt{d_k}}\right) {\textbf{V}} \end{aligned}$$

**Output Projection and Residual Connection:** The attention output is reshaped back to spatial dimensions [*B*, 2048, 8, 8] and processed through a final $1 \times 1$ convolution before adding the residual connection:

7 $$\begin{aligned} {\textbf{F}}_{\text {CSA}} = \text {Conv}_{1 \times 1}(\text {Attention}_{\text {out}}) + {\textbf{F}} \end{aligned}$$

The CSA module produces enhanced features ${\textbf{F}}_{\text {CSA}} \in {\mathbb {R}}^{B \times 2048 \times 8 \times 8}$ that serve as input to the subsequent CBAM module for further refinement.

**Fig. 3:**
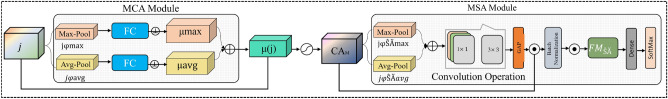
Conceptual Illustration of the Modified Dual Attention Module (MCBAM) showing the decomposition of $7 \times 7$ convolution into sequential $1 \times 1$ and $3 \times 3$ convolutions in the spatial attention component.

### Attention over attention

Attention mechanisms have been widely studied in visual intelligence, demonstrating significant effectiveness in improving feature representation by selectively concentrating on the most informative spatial and channel-wise cues^52^. We introduce the concept of attention over attention to further enhance the features through the strategic deployment of attention mechanisms at multiple stages. This approach involves the implementation of CBAM and its modified version (MCBAM) at two key positions within the network, significantly enhancing adaptability and performance. The first application of CBAM is positioned immediately after the CSA module, taking ${\textbf{F}}_{\text {CSA}}$ as input. This placement optimizes spatial and channel-wise attention post-CSA, enabling the network to make nuanced refinements by weighing the significance of features within both local and global contexts. The second instance involves MCBAM integrated following the RASA module, which further amplifies the network’s capabilities by refining multi-scale features.

#### Modified channel attention

The Channel Attention (CA) module captures relationships between features across different channels, where each feature map acts as a feature detector. Taking input from the CSA outputs (${\textbf{F}}_{\text {CSA}}$), the CA module comprises average pooling (Avg-P), maximum pooling (Max-P), and fully connected (FC) layers, as illustrated in Fig. 3. During training, not all convolution feature maps within a channel equally represent an object. Some channels excel at visualizing specific patterns while others excel differently. Our CA module incorporates both Max-P and Avg-P strategies, where Max-P sharpens focus on distinctive features while Avg-P offers a broader understanding. We apply these pooling strategies to the feature map spatial dimensions, resulting in:

8 $$\begin{aligned} & j\phi _{\text {avg}} = \text {Avg-P}({\textbf{F}}_{\text {CSA}}) \end{aligned}$$

9 $$\begin{aligned} & j\phi _{\text {max}} = \text {Max-P}({\textbf{F}}_{\text {CSA}}) \end{aligned}$$

Subsequently, we employ a single FC layer for each pooled feature, with shared parameters, followed by ReLU activation:

10 $$\begin{aligned} & \mu _{\text {max}} = \text {ReLU}(\text {FC}(j\phi _{\text {max}})) \end{aligned}$$

11 $$\begin{aligned} & \mu _{\text {avg}} = \text {ReLU}(\text {FC}(j\phi _{\text {avg}})) \end{aligned}$$

12 $$\begin{aligned} & \mu (j) = \mu _{\text {max}} \oplus \mu _{\text {avg}} \end{aligned}$$

The channel attention map is obtained through element-wise multiplication:

13 $$\begin{aligned} \text {CA}_{M} = \mu (j) \bigotimes {\textbf{F}}_{\text {CSA}} \end{aligned}$$

#### Modified spatial attention

The Spatial Attention (SA) module exploits spatial patterns to identify the most salient regions. Taking the channel attention output $\text {CA}_{M}$ as input, SA performs pooling operations across the channel dimension:

14 $$\begin{aligned} & j\phi _{\text {Sa-avg}} = \text {Avg-P}(\text {CA}_{M}) \end{aligned}$$

15 $$\begin{aligned} & j\phi _{\text {Sa-max}} = \text {Max-P}(\text {CA}_{M}) \end{aligned}$$

In our modified version, we replace the traditional $7 \times 7$ convolution with two sequential convolutions: a $1 \times 1$ convolution followed by a $3 \times 3$ convolution, as shown in Fig. 3. This modification reduces computational complexity while preserving essential spatial features:

16 $$\begin{aligned} M_{\text {Sa}} = f^{1 \times 1}(f^{3 \times 3}(j\phi _{\text {Sa-avg}} \oplus j\phi _{\text {Sa-max}})) \end{aligned}$$

The complete CBAM output is obtained as:

17 $$\begin{aligned} {\textbf{F}}_{\text {CBAM}} = M_{\text {Sa}} \bigotimes \text {CA}_{M} \end{aligned}$$

### Recursive atrous self-attention (RASA)

**Fig. 4:**
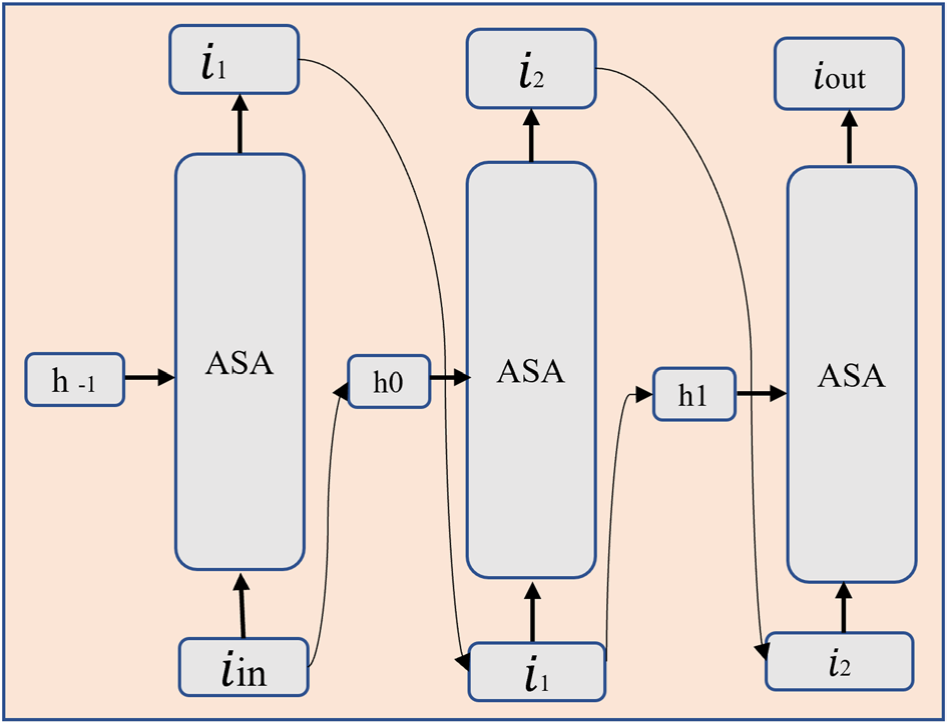
Overview of the RASA Module showing the recursive atrous self-attention mechanism with multi-scale context integration.

Following the CBAM module, the enhanced features ${\textbf{F}}_{\text {CBAM}}$ are processed through our proposed RASA module. RASA combines multi-scale context modeling with recursive processing for lightweight yet effective feature enhancement as shown in Fig. 4.

#### Atrous self-attention (ASA)

Multi-scale features are crucial for effective object detection and segmentation. Our ASA module integrates multi-scale information through atrous convolutions with different dilation rates. The query calculation is enhanced by transitioning from standard $1 \times 1$ convolution to:

18 $$\begin{aligned} Q = \sum _{\psi \in \{1, 3, 5\}} \text {SiLU}(\text {Conv}(\oint , P_{\alpha }^{\phi = 3}, r, G = D)) \end{aligned}$$

where the initial projection is:

19 $$\begin{aligned} \oint = \text {Conv}({\textbf{F}}_{\text {CBAM}}, P_{\alpha }^{\phi = 3}, r = 1, G = 1) \end{aligned}$$

and SiLU activation is defined as:

20 $$\begin{aligned} \text {SiLU}(n) = n \bigodot \text {sigmoid}(n) \end{aligned}$$

#### Recursive formulation

RASA extends ASA through recursive processing, following the recurrent network pipeline:

21 $$\begin{aligned} & i_{m+1} = \text {ASA}(f({\textbf{F}}_{\text {CBAM}}, H_{m-1})) \end{aligned}$$

22 $$\begin{aligned} & H_{m-1} = {\textbf{F}}_{m-1} \end{aligned}$$

23 $$\begin{aligned} & {\textbf{F}}_m = \text {ASA}(f({\textbf{F}}_{m-1}, H_{m-2})) \end{aligned}$$

where *m* represents the recursion step and *H* denotes the hidden state. The function $f({\textbf{F}}, H) = W_f {\textbf{F}} + u_f H$ with empirically optimal values $W_f = 1$ and $u_f = 1$. The recursion depth is limited to two to control computational costs, producing the final RASA output ${\textbf{F}}_{\text {RASA}}$.

### Modified CBAM (MCBAM)

Following the RASA module, we apply a MCBAM that incorporates the same architectural improvements as the first CBAM but operates on the multi-scale enhanced features from RASA. The MCBAM follows the same channel and spatial attention formulations but with the key modification of replacing $7 \times 7$ convolutions with sequential $1 \times 1$ and $3 \times 3$ convolutions for improved efficiency. The MCBAM takes ${\textbf{F}}_{\text {RASA}}$ as input and produces the final enhanced features ${\textbf{F}}_{\text {MCBAM}}$, which are then fed to the classification head for fire detection. This dual deployment of attention mechanisms (CBAM and MCBAM) at strategic positions creates a powerful attention-over-attention framework that progressively refines features from local context through global attention to multi-scale processing and final feature enhancement.

## Experimental results

This section provides an overview of the experimental setup, including descriptions of datasets, evaluation metrics, and training procedures. We perform a comprehensive empirical analysis that includes both quantitative performance metrics and qualitative analysis. The proposed network is supported by detailed ablation experiments that validate the architectural design.

### Experimental setup

Our experimental framework employs a high-performance computational environment consisting of an Intel Core i9 processor operating at 3.60 GHz. It is coupled with an NVIDIA GeForce RTX 4090 GPU equipped with 24 GB of RAM. The implementation is conducted using the PyTorch environment. All models are trained for 100 epochs across experimental configurations, with this epoch count empirically determined to ensure adequate convergence while maintaining generalization capability. All input imagery undergoes preprocessing to achieve standardized dimensions of $256 \times 256 \times 3$, ensuring consistent spatial resolution across the dataset. The training configuration uses essential hyperparameters, including a batch size of 16, to enhance memory utilization while ensuring the stability of gradient computation. It uses stochastic gradient descent (SGD) optimization with a learning rate of $1 \times 10^{-3}$ and a momentum coefficient of 0.9 to facilitate convergence acceleration.

### Datasets

**Fig. 5:**
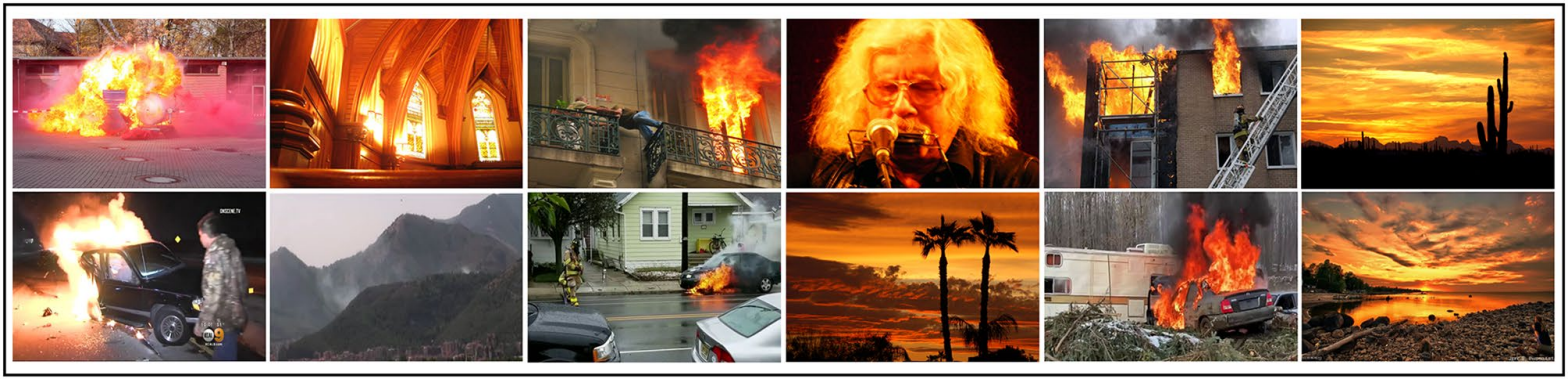
Exemplary Images from the datasets that display multi-dimensional views and challenging fire/non-fire scenarios samples.

We evaluate our proposed AEFRN architecture on two established benchmark datasets: FD^48^ and BoWFire^24^. Both datasets follow an 80/20 train-test split for consistent evaluation. Representative samples are shown in Fig. 5. The **FD dataset** contains 50,000 balanced images (25,000 fire and 25,000 non-fire) aggregated from multiple sources, including Foggia and BoWFire repositories. This large-scale dataset provides diverse fire scenarios with varying flame characteristics, lighting conditions, and environmental settings, making it suitable for robust model training. The **BoWFire dataset** comprises 226 images (107 fire and 119 non-fire) designed for challenging binary classification scenarios. Despite its smaller size, this dataset features complex visual conditions, including subtle flames, fire-like objects, and cluttered backgrounds.

### Evaluation metrics

We evaluate the proposed AEFRN architecture using four standard metrics: Precision, Recall, Accuracy, and F1-score. These metrics are widely adopted in fire detection literature^33,48^ and provide a comprehensive assessment of detection precision, sensitivity, and overall classification performance across balanced and imbalanced scenarios. The mathematical formulations of these evaluation criteria follow conventional definitions established in the referenced studies.

### Performance evaluation of AEFRN

We perform an extensive empirical analysis to assess the efficacy of our proposed AEFRN compared to existing SOTA methodologies. This comprehensive evaluation includes a quantitative assessment using the previously specified metrics, along with a qualitative examination through visual analysis.

**Table 1:** Quantitative evaluation of our network and competing techniques using the two datasets. The Italic values highlight cases where our network achieves top performance, while the Bold values indicate the competing network ranks second-best performance.

Methods	FD				BoWFire			
	Pre	Rec	F1	Acc	Pre	Rec	F1	Acc
FD-GCM^53^	-	-	-	-	55.00	54.00	54.00	-
FFD-ANN^54^	71.10	73.20	72.10	71.10	-	-	-	-
EFD-IP^22^	75.00	15.00	25.00	-	-	-	-	-
FPC^9^	52.00	99.90	68.40	53.90	-	-	-	-
BowFire^24^	-	-	-	-	51.00	65.00	67.00	-
LW-CNN^55^	82.00	81.00	81.00	81.00	86.00	78.00	77.00	79.00
ResNetFire^33^	-	-	-	-	-	-	-	92.50
EFDNet^48^	93.50	97.40	95.40	95.30	81.81	83.00	81.85	83.33
DFAN-Comp^15^	95.50	96.30	95.90	95.70	94.30	92.00	93.10	93.00
ConvNeXt-TSA^56^	97.99	96.72	97.33	98.00	94.48	93.31	93.80	94.21
DFAN^15^	96.00	97.00	96.00	96.17	95.00	94.00	95.00	95.00
MAFire-Net^56^	98.68	98.68	98.68	99.00	97.05	98.15	97.77	97.82
AEFRN (Ours)	*98.95*	*98.82*	*98.88*	*99.11*	*97.88*	*98.24*	*97.94*	*97.98*

#### Quantitative analysis

We conducted a comprehensive empirical evaluation comparing our proposed AEFRN with multiple SOTA approaches, including both conventional ML and DL frameworks. The evaluation was conducted on two datasets, FD^48^ and BoWFire^24^, with results presented in Table 1. Throughout both datasets and across all evaluation metrics, AEFRN consistently showed greater performance. On the FD, the network achieved an overall accuracy of 99.11%. On the BoWFire dataset, AEFRN maintained similarly high performance, with precision reaching 97.88%, recall at 98.24%, F1-score at 97.94%, and accuracy of 98.11%. When compared to traditional ML-based models, AEFRN offers significant improvements. These gains highlight the model’s effectiveness and robustness in large-scale detection tasks. In the context of the BoWFire, AEFRN achieved an accuracy of 97.98 %, significantly outperforming other approaches. This illustrates AEFRN’s robust generalization ability across datasets and its significant advantage over traditional methods that depend on handcrafted features. When evaluated on the FD, AEFRN exhibits enhanced performance with a 0.27 % gain in precision, 0.14 % improvement in recall, 0.20 % increase in F1-score, and 0.11 % advancement in accuracy relative to MAFire-Net. For the BoWFire benchmark, AEFRN demonstrates superior results compared to MAFire-Net, achieving a 0.83 % precision enhancement, 0.09 % recall improvement, 0.17 % F1-score increase, and 0.29 % accuracy gain. These experimental outcomes confirm that our model’s hierarchical attention-over-attention methodology successfully integrates detailed local feature extraction with comprehensive contextual analysis, facilitating dependable fire detection performance in challenging operational environments.

#### Qualitative comparisons

**Fig. 6:**
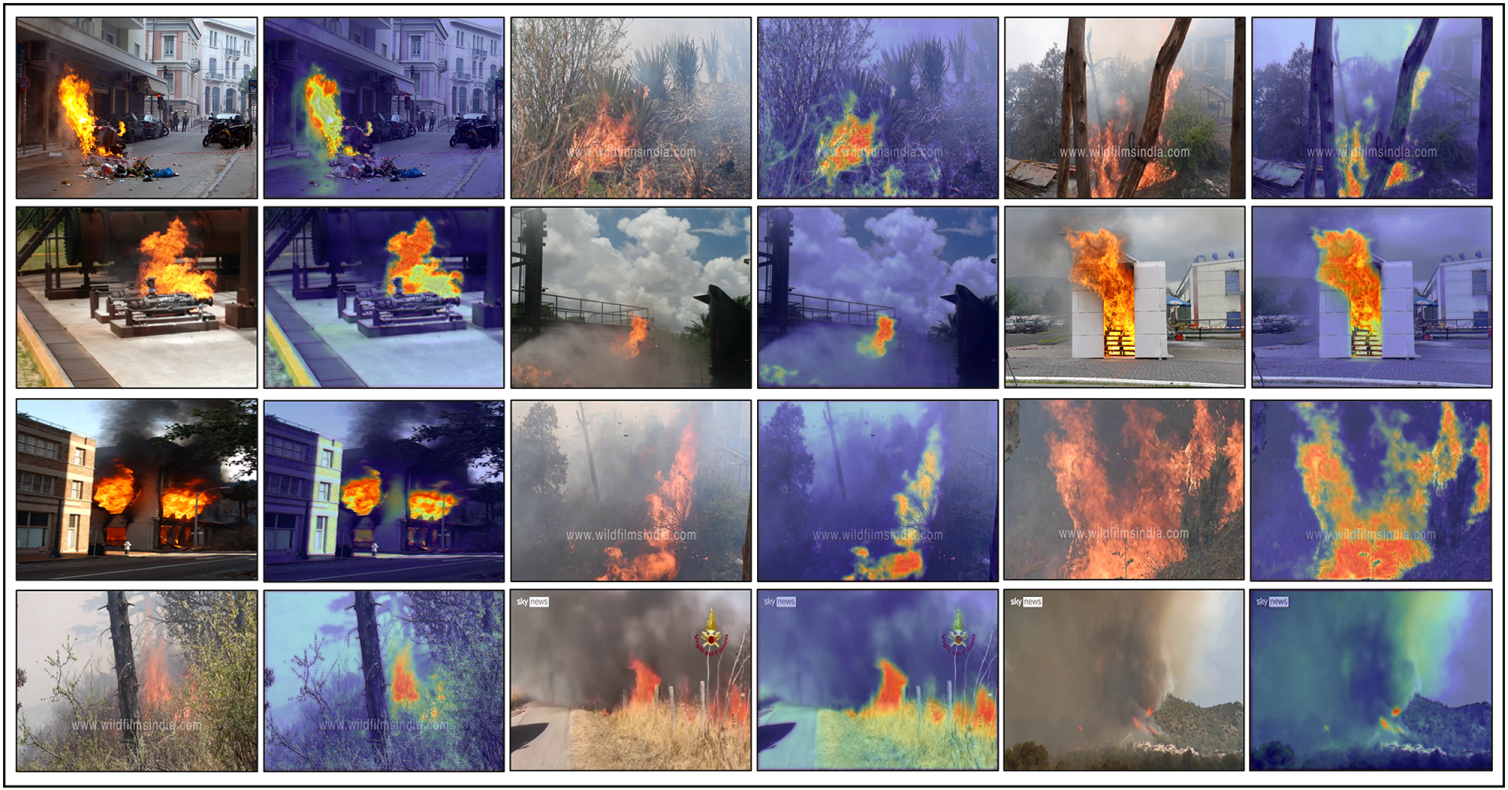
Visual interpretation of AEFRN through Grad-CAM analysis on challenging fire detection cases. Input test images are paired with their respective attention maps, revealing the network’s focus on fire-related regions.

As demonstrated in Fig. 6, our network exhibits remarkable proficiency in identifying and precisely localizing fire regions within complex environmental contexts. The visual examination employs attention visualization maps generated through AEFRN’s hierarchical attention framework, which highlight the most discriminative spatial regions. Qualitative analysis demonstrates the model’s superior interpretability and accurate localization across diverse scenarios. Particularly noteworthy is the network’s ability to maintain accurate fire detection in scenarios with heavy smoke, varied flame intensities, and cluttered backgrounds where traditional color-based or motion-based approaches often fail. The attention visualizations reveal that AEFRN’s progressive attention mechanism successfully captures both local fire characteristics (flame textures, color patterns) and global contextual information (environmental context, spatial relationships). The consistent quality of attention maps across diverse scenarios validates the effectiveness of our attention-over-attention framework in learning discriminative fire representations that generalize effectively to unseen environments and fire conditions.

### Ablation study

We conducted comprehensive ablation studies to identify the most effective AEFRN configuration and validate our design choices. These studies involved testing various backbone networks, module combinations, data augmentation strategies, and hyperparameter configurations to assess the performance of our network. Detailed information regarding the experimental ablations can be found in Tables 2, 3, 4 and 5, with further elaboration provided in the following subsections.

#### Backbone network analysis

To validate our choice of ResNet50 as the backbone with our proposed network, we conducted a comprehensive comparison across six different backbone architectures. It is noteworthy that the remaining five models were evaluated based on their performance using pretrained weights. As shown in Table 2, ResNet50 with the proposed network consistently delivers superior performance across both datasets. On the FD dataset, it achieves the highest accuracy of **99.11%**, precision of **98.95%**, recall of **98.82%**, and F1-score of **98.88%**. Similarly, on the BoWFire dataset, the network with ResNet50 attains exceptional performance with accuracy of **97.98%**, precision of **97.88%**, recall of **98.24%**, and F1-score of **97.94%**. The superior performance of the best results can be attributed to its optimal balance between feature extraction capability and computational efficiency, along with its proven effectiveness in capturing hierarchical representations essential for fire detection tasks.

**Table 2:** Backbone network comparison for AEFRN architecture. ResNet50 achieves the best performance with the proposed network.

Backbone	FD				BoWFire			
	Acc	Pre	Rec	F1	Acc	Pre	Rec	F1
VGG16	94.23	93.45	95.12	94.28	92.87	91.65	94.25	92.93
VGG19	94.67	93.85	95.68	94.76	93.24	92.15	94.78	93.45
ResNet34	95.89	95.12	96.85	95.98	94.75	93.92	95.86	94.88
ResNet101	96.45	95.78	97.25	96.51	95.34	94.58	96.45	95.51
DenseNet121	95.67	94.89	96.58	95.73	94.52	93.78	95.65	94.71
ResNet50	**99.11**	**98.95**	**98.82**	**98.88**	**98.11**	**97.88**	**98.24**	**97.94**

#### Effect of module combinations

In our thorough evaluation of AEFRN, we examined various architectural configurations to identify the best module arrangement. These included combinations such as CSA with RASA, dual RASA modules, and CBAM-enhanced variants. As shown in Table 3, the final configuration **CSA+CBAM+RASA+MCBAM** consistently delivered superior results. Specifically, on the **FD** dataset, it achieved the highest accuracy of **99.11%**, precision of **98.95%**, recall of **98.82%**, and F1-score of **98.88%**. Similarly, on the **BoWFire** dataset, this configuration attained an accuracy of **97.98%**, precision of **97.88%**, recall of **98.24%**, and F1-score of **97.94%**. These results confirm the effectiveness of our complete AEFRN configuration, showcasing the synergistic effect of combining convolutional self-attention, channel-spatial attention, and recursive atrous self-attention for robust fire detection across diverse datasets.

**Table 3:** Ablation study of module combinations in AEFRN. The values highlighted in bold indicate the top performance achieved by the complete AEFRN configuration.

Module Configuration	FD				BoWFire			
	Acc	Pre	Rec	F1	Acc	Pre	Rec	F1
ResNet50 + CSA	89.25	87.85	90.15	88.98	87.92	86.45	89.25	87.84
ResNet50 + CSA + RASA	91.45	90.12	92.85	91.47	90.68	89.35	91.95	90.64
ResNet50 + CSA + CBAM	92.78	91.56	93.95	92.75	91.89	90.78	93.15	91.96
ResNet50 + CSA + RASA + RASA	94.23	93.15	95.45	94.29	93.45	92.25	94.75	93.49
ResNet50 + CSA + CBAM + RASA	95.67	94.85	96.68	95.76	94.89	93.95	95.98	94.96
ResNet50 + CBAM + RASA + CBAM	96.89	95.98	97.85	96.91	95.78	94.86	96.85	95.85
ResNet50 + CSA + RASA + CBAM	97.34	96.45	98.25	97.34	96.52	95.68	97.45	96.56
ResNet50 + CSA + CBAM + RASA + MCBAM	**99.11**	**98.95**	**98.82**	**98.88**	**98.11**	**97.88**	**98.24**	**97.94**

#### Data augmentation analysis

Data augmentation plays a crucial role in enhancing model robustness and generalization, particularly when working with limited and imbalanced datasets. To investigate its impact, we conducted a series of experiments using diverse augmentation techniques for fire detection. Table 4 summarizes the performance of AEFRN across two benchmark datasets (**FD** and **BoWFire**) using several augmentation strategies. Without augmentation, the AEFRN model already delivers strong performance, achieving an accuracy of 96.78% and 95.23% on the FD and BoWFire datasets, respectively. However, we observe that applying augmentation leads to consistent improvements across all metrics—accuracy, precision, recall, and F1 Score. Among individual techniques, **color jittering** proved most effective, improving the F1-score from 96.45% to 97.89% on the FD dataset and from 95.01% to 96.78% on BoWFire. This suggests that simulating variations in fire color and brightness enhances the model’s ability to distinguish between real flames and fire-like patterns in complex backgrounds. Furthermore, combining multiple augmentations yields even more pronounced gains. The trio of **random flip**, **random crop**, and **color jittering** leads to a significant performance boost, achieving 98.34% F1-score on FD and 97.25% on BoWFire. These results indicate that the combined effect of spatial and photometric variations enables the model to learn more discriminative and invariant features. Our proposed augmentation strategy, which selectively integrates all augmentation techniques using fire-specific parameter tuning and a strategically optimized order, yields the best results across both datasets. Specifically, it achieves an accuracy of **99.11%** on FD and **97.98%** on BoWFire, along with leading scores in precision, recall, and F1.

**Table 4:** Effect of different data augmentation techniques on AEFRN Performance. The results highlighted in bold show the best performance achieved by our proposed network.

Augmentation Strategy	FD				BoWFire			
	Acc	Pre	Rec	F1	Acc	Pre	Rec	F1
No Augmentation	96.78	95.85	97.35	96.45	95.23	94.15	95.95	95.01
Random Flip	97.45	96.65	98.15	97.39	96.12	95.25	96.88	96.06
Random Rotation	97.23	96.35	97.95	97.14	95.86	94.89	96.65	95.76
Random Crop	97.68	96.89	98.35	97.61	96.45	95.58	97.25	96.41
Color Jittering	98.12	97.35	98.68	97.89	96.89	95.95	97.68	96.78
Gaussian Noise	97.02	96.15	97.78	96.96	95.68	94.75	96.45	95.59
Flip + Rotation	97.89	97.15	98.45	97.79	96.68	95.85	97.45	96.64
Flip + Crop + Color	98.45	97.78	98.95	98.34	97.35	96.58	98.12	97.25
Rotation + Color + Noise	98.23	97.56	98.75	98.15	97.15	96.35	97.85	97.09
All Augmentations	98.78	98.15	99.25	98.69	97.78	96.95	98.45	97.69
Proposed Strategy	**99.11**	**98.95**	**98.82**	**98.88**	**98.11**	**97.88**	**98.24**	**97.94**

#### Impact of dilation rates in RASA

To evaluate efficacy and establish the optimal dilation parameter configuration, we examined multiple dilation arrangements. Table 5 presents the comparative results across four essential evaluation criteria. Employing a singular dilation rate $\{1\}$, equivalent to conventional convolution operations, produces comparatively modest performance levels, achieving F1-scores of 97.42% and 96.48% on FD and BoWFire datasets, respectively. Extending to a dual-parameter configuration {1, 3} enhances performance, achieving F1-scores of 98.18% and 97.56%. Our recommended configuration $\{1, 3, 5\}$ achieves superior overall performance with F1-scores of **98.88%** on FD and **97.94%** on BoWFire. Incorporating an extended dilation arrangement $\{1, 3, 5, 7\}$ yields marginal improvements on FD while providing negligible enhancement on BoWFire, indicating diminishing performance benefits beyond optimal dilation coverage. Conversely, omitting the minimal dilation parameter in configuration $\{3, 5, 7\}$ moderately degrades performance. These experimental findings confirm that the $\{1, 3, 5\}$ parameter arrangement delivers the optimal balance between scale diversity and feature representation capacity.

**Table 5:** Effect of different dilation rate configurations in RASA. The values in bold indicate the optimal performance achieved by the proposed configuration.

Dilation Rates	FD Dataset				BoWFire Dataset			
	Acc	Pre	Rec	F1	Acc	Pre	Rec	F1
{1}	97.35	96.92	97.88	97.42	96.18	95.85	96.92	96.48
{1, 3}	98.29	97.75	98.61	98.18	97.52	97.12	97.98	97.56
{1, 3, 5} (Proposed)	**99.11**	**98.95**	**98.82**	**98.88**	**98.11**	**97.88**	**98.24**	**97.94**
{1, 3, 5, 7}	99.05	98.86	98.76	98.95	98.08	97.82	98.23	98.02
{3, 5, 7}	98.12	97.58	98.36	98.05	96.91	96.28	97.54	96.91

## Discussion and insights

The experimental findings demonstrate that AEFRN delivers superior performance through its innovative attention-based architecture. The network’s effectiveness originates from strategic design choices that address limitations in existing literature, particularly in complex scenarios. The proposed multi-layered attention mechanism represents a significant departure from conventional single-scale approaches by establishing a hierarchical processing pipeline. Through the effective combination of CSA, RASA, and the enhanced CBAM, AEFRN makes a progressive feature refinement process that adaptively focuses on fire-relevant spatial regions. The hierarchical design proves particularly valuable in scenarios with cluttered backgrounds or visually similar non-fire elements. The RASA component’s multi-scale dilated convolution strategy allows for effective contextual analysis across different spatial resolutions. This capability is essential due to the diverse nature of fire phenomena, which range from small ignition points to large flame structures. AEFRN’s consistent performance and testing conditions showcase strong generalization capabilities. The comprehensive data augmentation strategy, along with the attention-driven modules, facilitates the extraction of invariant fire properties that stay stable across diverse environmental conditions. Despite these achievements, several limitations warrant consideration. Extreme environmental conditions involving severe illumination variations or significant occlusion may impact the localization accuracy. The current emphasis on analyzing static images neglects the importance of maintaining temporal consistency in video sequences, which remains unexplored. Furthermore, the network’s ability to detect atypical or synthetic fire scenarios warrants additional research. Although the model demonstrates promising computational efficiency for edge applications, empirical testing on severely resource-limited IoT platforms is essential to verify real-time operational feasibility.

## Conclusion and future work

This research presents the Attention-Enhanced Fire Recognition Network (AEFRN), an innovative visual attention-based architecture for fire detection that demonstrates superior performance through sophisticated attention methodologies. Our proposed framework integrates Convolutional Self-Attention (CSA) to synthesize global and local feature representations, implements Recursive Atrous Self-Attention (RASA) with strategically configured dilation parameters for multi-resolution contextual extraction, and incorporates an enhanced CBAM to strengthen feature discrimination capabilities. Comprehensive experimental validation across the FD and BoWFire benchmark collections establishes substantial performance advantages relative to contemporary methodologies. Detailed ablation studies affirm our architectural decisions, confirming that ResNet50 is the ideal backbone while highlighting the synergistic advantages of the overall configuration. The proposed data augmentation strategy and three-level dilation configuration further enhance detection robustness across diverse fire scenarios. AEFRN’s balanced design ensures computational efficiency suitable for resource-constrained environments while maintaining exceptional accuracy, making it highly applicable for real-world fire detection systems. In the future, we aim to focus on extending AEFRN for aerial fire monitoring applications, developing lightweight variants for IoT deployment, and integrating temporal modeling for video-based detection. We also plan to explore federated learning approaches for distributed fire detection networks and multi-class fire classification capabilities to enhance emergency response systems.

## Data Availability

The Bowfire dataset used in this study is publicly available at https://www.kaggle.com/datasets/malligasenthil/bowfire. The FD dataset analyzed in this study was introduced in a previously published work in *IEEE Transactions on Image Processing* (https://doi.org/10.1109/TIP.2020.3016431) and can be made available upon reasonable request to the corresponding author.
